# The role of T cell trafficking in CTLA-4 blockade-induced gut immunopathology

**DOI:** 10.1186/s12915-020-00765-9

**Published:** 2020-03-17

**Authors:** Shashuang Zhang, Wenhua Liang, Lingjie Luo, Shan Sun, Feng Wang

**Affiliations:** 1 Research Center of Translational Medicine, Shanghai Children’s Hospital, Shanghai Jiao Tong University School of Medicine, Shanghai, China; 2 The Center for Microbiota and Immunological Diseases, Shanghai General Hospital, Shanghai Institute of Immunology, Department of Immunology and Microbiology, Shanghai Jiao Tong University School of Medicine, Shanghai, China

**Keywords:** CTLA-4 blockade, Colitis, CD4 T cells, Trafficking-blocking antibody

## Abstract

**Background:**

Immune checkpoint inhibitor (ICPI) can augment the anti-tumour response by blocking negative immunoregulators with monoclonal antibodies. The anti-cytotoxic T lymphocyte-associated antigen-4 (CTLA-4) antibody is the first ICPI which has shown remarkable benefits in the clinical treatment of cancers. However, the increased activity of the immune system also causes some side effects called immune-related adverse events (irAEs). Colitis is one of the most common irAEs related to anti-CTLA-4 immunotherapy.

**Results:**

We identified that CD4^+^ T cells were the primary responders in CTLA-4 blockade and that the expansion of gut-homing CD4^+^ T cells by anti-CTLA-4 therapy was independent of CD103. We used dextran sulfate sodium (DSS)-induced colitis mice as our model and tested the possibility of using a trafficking-blocking antibody to treat anti-CTLA-4 antibody-induced irAEs. We found that blocking T cell homing increased colitis severity in the context of CTLA-4 blockade and that gut-trafficking blockade had different effects on different Th subsets and could facilitate the proliferation of Th17 cells in the lamina propria (LP).

**Conclusions:**

Our data reveals the fundamental mechanism underlying trafficking-blocking antibody therapy for CTLA-4 blockade-induced colitis and provide a caution in regard to apply trafficking-blocking antibody treatment under CTLA-4 blockade condition.

## Background

Cancer immunotherapy has become a powerful method to treat malignant tumours in addition to traditional methods such as surgery, radiotherapy and chemotherapy [1–3]. As one kind of immunotherapy, immune checkpoint inhibitor (ICPI) therapy has shown significant survival benefits in a wide range of cancers, including melanoma, lung cancer, kidney cancer and head and neck cancer [1, 4, 5]. ICPIs are a group of monoclonal antibodies that can reverse the immune tolerance mediated by cancer cells and generate a long-term anti-tumour immune response [6]. As the first ICPI, ipilimumab, which can block cytotoxic T lymphocyte-associated antigen-4 (CTLA-4), was approved by the US Food and Drug Administration (FDA) for the treatment of metastatic melanoma in 2011 [7, 8]. CTLA-4 is a negative regulator of co-stimulation that can control the initial activation and proliferation of T cells [9]. The blockade of CTLA-4 with anti-CTLA-4 antibodies can enhance the immune response to tumours [10, 11]. However, the augmented immune response enabled by these agents also leads to a particular group of side effects called immune-related adverse events (irAEs) [12–16]. The distribution of grade 3–5 irAEs across all tumour types in the main clinical trial testing anti-CTLA-4 antibodies indicates that these side effects mainly occur in the gastrointestinal tract [17]. In an analysis of 193 fatal ICPI-related toxic events caused by anti-CTLA-4 antibodies, 135 event (~ 70%) fatalities were related to colitis [18].

When somewhere in the body is attacked, the immune system will recruit various kinds of lymphocytes to the site to defend against pathogens. Hence, the trafficking and localization of lymphocytes is critical in tissue inflammation. The process of T cell homing to the gut requires β7-containing integrins. β7 is one of the integrin subunits; it noncovalently associates with the α4 subunit to become α4β7 integrin, which functions as a transmembrane cell adhesion receptor and is expressed on the surface of T cells. α4β7 integrin binds to its ligand mucosal addressin cell adhesion molecule-1 (MAdCAM-1), which is constitutively expressed on high endothelial venules, to mediate T cell extravasation from the blood to the gut mucosal tissues of the gut-associated lymphoid tissue (GALT), such as Peyer’s patches (PPs), mesenteric lymph node (MLN) and lamina propria (LP) [19, 20]. Currently, trafficking-blocking antibodies have already become a novel class of immune intervention therapeutics in the clinic [21]. There are some trafficking-blocking antibodies that have been approved by the FDA or are in the process of being evaluated in clinical trials. For example, vedolizumab acts on gut-trophic α4β7 integrin and has been approved to treat inflammatory bowel disease (IBD) [22, 23]; etrolizumab, which is in the clinical trials for ulcerative colitis, can target and bind to the β7 subunit [24]. Clinical case series suggest that vedolizumab is effective for treating steroid-refractory enterocolitis caused by ICPIs [25].

In this study, we chose the dextran sulfate sodium (DSS)-induced colitis mouse model, which is one of the most common models of chemically induced colitis in mice and is used to mimic human inflammatory bowel disease [26]. We combined the DSS model with the administration of an anti-CTLA-4 blocking antibody to investigate the mechanism by which CTLA-4 blockade influences intestinal inflammation [27]. In particular, we tested the possibility of treating irAEs induced by CTLA-4 blockade with a gut-homing trafficking-blocking antibody by dissecting the molecular and cellular mechanisms underlying these processes.

## Results

### Gut-homing CD4^+^ T cells are primary responders in CTLA-4 blockade

**Fig. 1:**
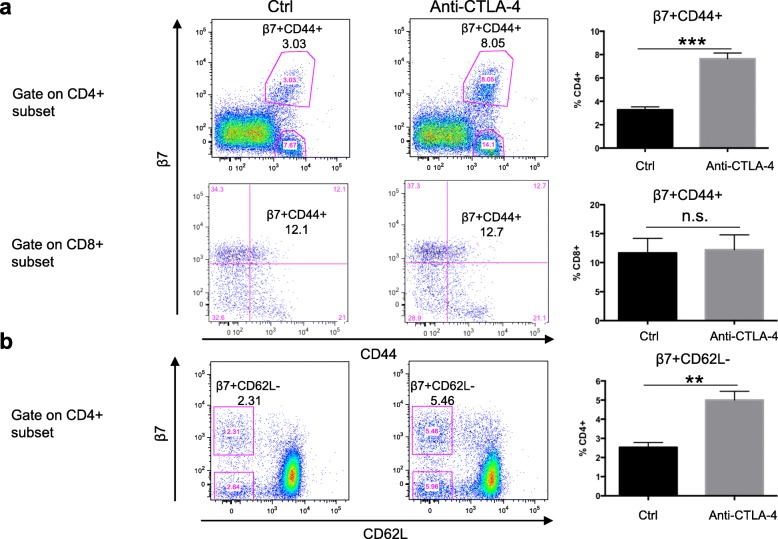
Peripheral blood CD4^+^ T cells are responsible for the anti-CTLA-4 antibody effect. Flow cytometric analysis of gut-homing T cells in the blood 10 days after isotype antibody (Ctrl) or anti-CTLA-4 mAb treatment. **a** Representative flow plots depicting activated gut-homing T cells (β7^+^CD44^+^) (left) and quantification of activated gut-homing T cells (right). The upper panel was gated from live CD4^+^ subset and the lower gated from live CD8^+^ subset. **b** Representative flow plots depicting activated gut-homing CD4^+^ T cells (β7^+^CD62L^−^) (left) and quantification of activated gut-homing T cells (right). The numbers indicate the percentage of total cells in the indicated gate. The right bar graph shows a summary of the data for 5 mice in each group (mean ± SEM). For all graphs: **P < 0.01 and ***P < 0.001

To study the effect of CTLA-4 blockade on gut-homing T cells, we treated wild-type (WT) C57BL/6 mice with an anti-CTLA-4 antibody or isotype control antibody and examined T cells in the peripheral blood. Here, we used two indicators, CD44 and β7; CD44 is a T cell activation marker, and β7 indicates gut-homing T cells. In the CD4^+^ subset, the percentage of activated gut-homing CD4^+^ T cells was increased over 2 times after anti-CTLA-4 antibody treatment compared with control treatment, while there was no significant change in blood CD8^+^ T cells (Fig. 1a). The other T cell activation marker, CD62L, also showed a similar pattern in the CD4^+^ subset (Fig. 1b). We also detected the surface-expressed homing marker α4, and the number of α4^+^CD44^+^ blood CD4 T cells also increased after CTLA-4 blockade, which was consistent with our β7 expression data (Additional file 1: Figure S1). The staining result of isotype controls for both anti-β7 and anti-CD44 antibodies in the Additional file 2: Figure S2. The gene expression profile from the Immunological Genome (ImmGen) database showed that the CTLA-4 expression level was significantly higher in the CD4^+^ subset (especially in Treg cells) than in the CD8^+^ subset (Additional file 3: Figure S3) [28]. Therefore, we can infer that gut-homing activated CD4^+^ T cells are the major responder population in anti-CTLA-4 blockade, at least in the initial stage.

### CTLA-4 blockade increases gut-homing CD4 T cell numbers independent of CD103

**Fig. 2:**
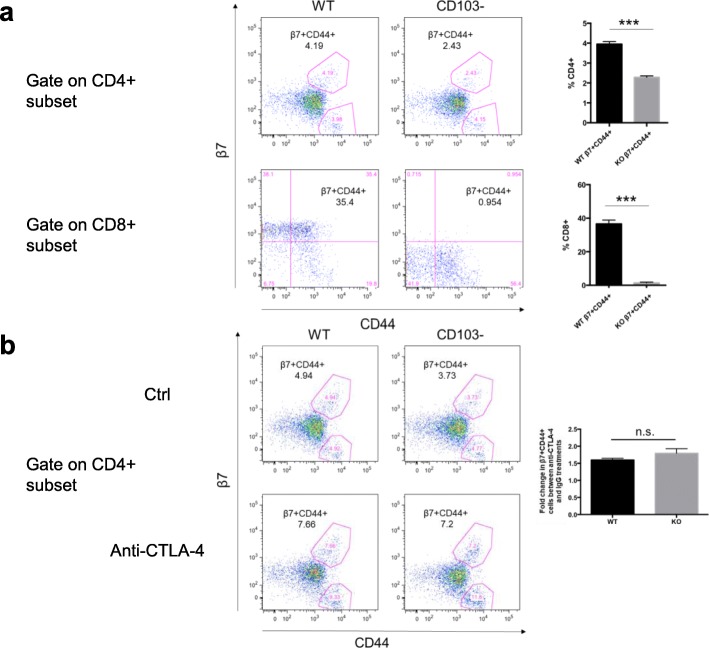
Increase in gut-homing CD4^+^ T cell numbers induced by anti-CTLA-4 therapy is independent of CD103. **a** Knocking out CD103 decreased β7 expression in blood CD4^**+**^ and CD8^**+**^ T cells. Flow cytometric analysis of activated gut-homing T cells in the blood from WT and CD103 KO mice. Representative flow plots depicting activated gut-homing T cells (β7^+^CD44^+^) (left) and quantification of activated gut-homing T cells (right). The upper profiles were gated on live CD4^**+**^ T cells, and the lower profiles were gated on live CD8^**+**^ T cells. **b** The increase in gut-homing CD4^**+**^ T cell numbers by anti-CTLA-4 therapy was independent of CD103. Flow cytometric analysis of activated gut-homing CD4^**+**^ T cells in the blood 10 days after isotype antibody (Ctrl) (upper profiles) or anti-CTLA-4 mAb (lower profiles) treatment from WT and CD103 KO mice. The left profiles are representative flow plots depicting activated gut-homing T cells (β7^+^CD44^+^), and the right bar graph shows the fold change in activated gut-homing T cells (β7^+^CD44^+^) between the anti-CTLA-4 group and the IgG group. There were 5 mice in each group, data represent mean ± SEM, ***P < 0.001

From a previous study, we already know that CD103^+^ dendritic cells (DCs) are the main regulators of intestinal T cell homing. When naïve T cells encounter relevant antigenic peptide-MHC complexes, they become activated, and CD103^+^ DCs induce the upregulation of α4β7 and CCR9 expression on the T cell surface, which results in the interactions with the corresponding ligands that help the T cells migrate to sites of inflammation in the gut and execute their function [29]. To determine the underlying mechanism of gut-homing T cell expansion in the blood after CTLA-4 blockade, we first hypothesised that CTLA-4 blockade influences the classic gut-imprinting mechanism, which promotes the expression of α4β7. To test this possibility, we detected activated gut-homing T cells in CD103 knock-out (KO) mice. We found that the percentage of blood β7^+^CD44^+^ cells decrease in the CD103 KO mice compared to their WT counterparts for both CD4^+^ and CD8^+^ T cell populations (Fig. 2a), which indicates that CD103 is responsible for β7 expression and gut-homing imprinting in T cells. Surprisingly, when we treated mice with an anti-CTLA-4 antibody, the fold increase in gut-homing CD4^+^ T cells in CD103 KO mice was the same as that in WT mice (Fig. 2b). Therefore, we concluded that the increase in the number of gut-homing CD4^+^ T cells induced by anti-CTLA-4 therapy was the result of proliferation by pre-existing gut-homing T cells and did not require an additional CD103-dependent imprinting process.

### Gut tissue damage drives T cell homing under CTLA-4-blockade conditions

**Fig. 3:**
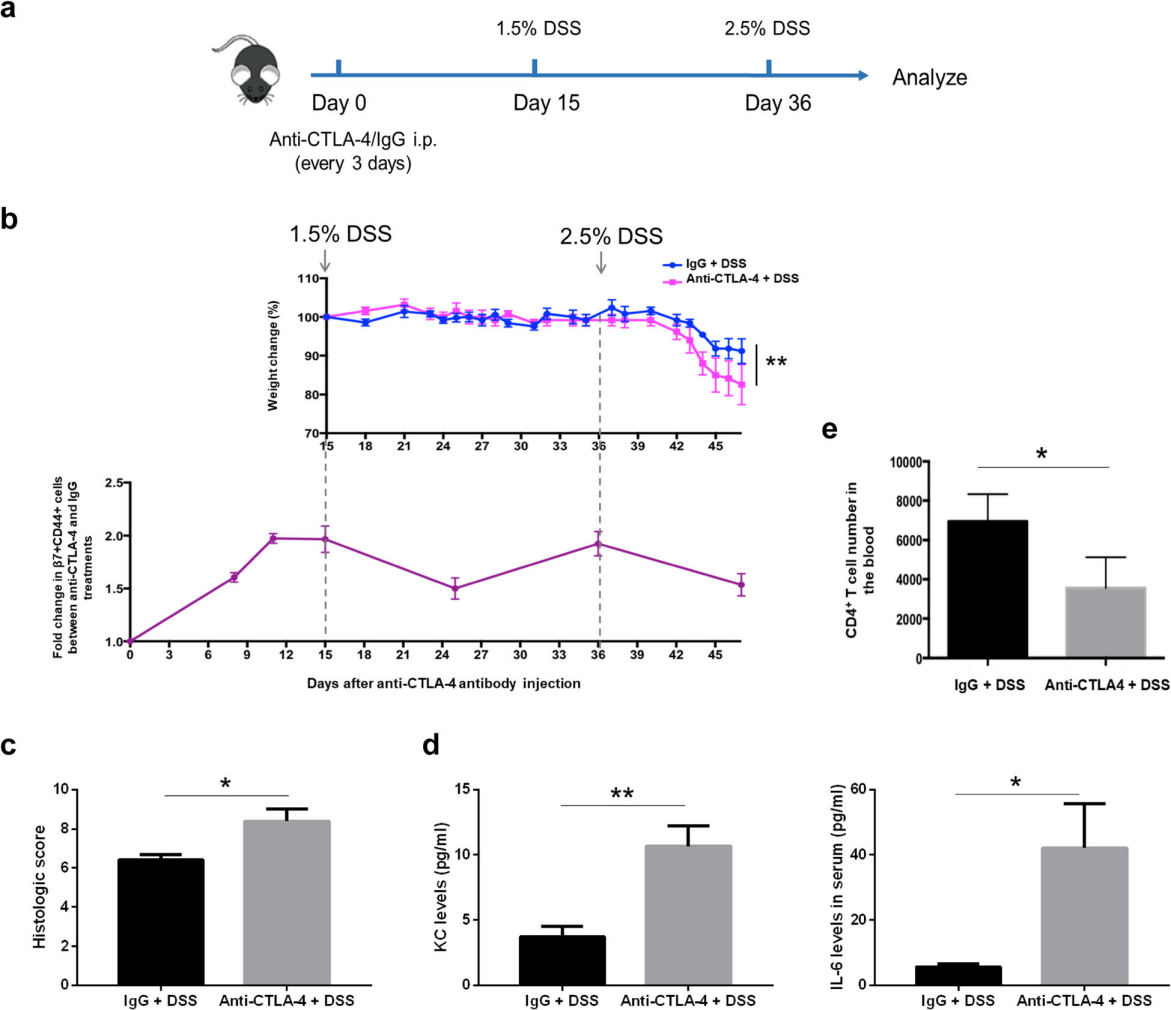
Anti-CTLA-4 antibody- and DSS-induced inflammation drive gut-homing T cell extravasation from the blood. **a** The experimental design for the whole process. WT mice were treated with anti-CTLA-4 mAb or isotype control antibody every 3 days and given 1.5% DSS beginning on day 15 and 2.5% DSS beginning on day 36, each time lasts 4 days and then changed back to water. **b** Percent of initial weight of mice receiving an IgG isotype control (Iso Ctrl) or anti-CTLA-4 mAb (upper profile). The dynamic change in blood β7^+^CD44^+^CD4^**+**^ T cells is shown as the fold change in β7^+^CD44^+^ cells between the anti-CTLA-4 antibody-treated group and the IgG-treated group (lower profiles). There were 5 mice in each group. The data are shown as the mean and SEM determined by two-way ANOVA with Sidak’s correction for multiple comparisons, **P < 0.01. **c** The histological score of mice receiving the IgG isotype control (Iso Ctrl) or the anti-CTLA-4 mAb. Colon samples were collected at day 47. **d** Concentrations of KC and IL-6 in the serum of mice receiving the IgG isotype control (Iso Ctrl) or the anti-CTLA-4 mAb (day 47). **e** Summary of the blood CD4^**+**^ T cell numbers in the mice on day 47. *n* = 5 per group, data represent mean ± SEM. For all graphs: *P < 0.05 and **P < 0.01

Next, we used the DSS colitis model to test the effect of tissue damage on blood gut-homing T cells under CTLA-4-blockade conditions. We divided WT mice into two groups: one group was injected with an anti-CTLA-4 antibody every 3 days, and the other group was injected with an IgG isotype control. We then treated the mice with 1.5% DSS for 4 days beginning on day 15, and on day 36, we treated the mice again with 2.5% DSS (Fig. 3a). Consistent with our previous data [27], when we increased the concentration of DSS to 2.5%, the group treated with CTLA-4 blockade exhibited a greater decline in weight than the isotype control group, which indicated that inflammation was more severe under CTLA-4-blockade conditions (Fig. 3b, upper panel). Histopathological scores of colon sections from anti-CTLA-4 mAb-treated mice were also significantly worse than the group of mice that received isotype control antibodies (Fig. 3c). Serum levels of inflammatory cytokines, KC and IL6, were also increased in anti-CTLA-4 mAb-treated mice (Fig. 3d). At the same time, we analysed the dynamic change in blood gut-homing CD4^+^ T cells through monitoring the fold change in β7^+^CD44^+^ subsets in the anti-CTLA-4 antibody-treated group versus the isotype control-treated group. We found that the fold change in gut-homing T cells in the blood increased gradually after anti-CTLA-4 antibody injection due to the proliferation of gut-homing CD4^+^ T cells driven by CTLA-4 blockade. When we treated mice with DSS to induce intestinal inflammation, the fold change declined correspondingly. The pattern changed along with the occurrences of DSS-induced intestinal damage during the whole process (Fig. 3b, lower panel). Moreover, we confirmed that the CD4^+^ T cell number in the blood of anti-CTLA-4 group mice at the end of the experiment was lower than that in the blood of control group mice (Fig. 3e), which correlated with severe T cell infiltration into the damaged intestinal tissue. These data demonstrated that anti-CTLA-4-blockade- and DSS-induced intestinal inflammation drove gut-homing T cell extravasation from the blood.

### Blocking T cell homing increases colitis severity under CTLA-4-blockade conditions

**Fig. 4:**
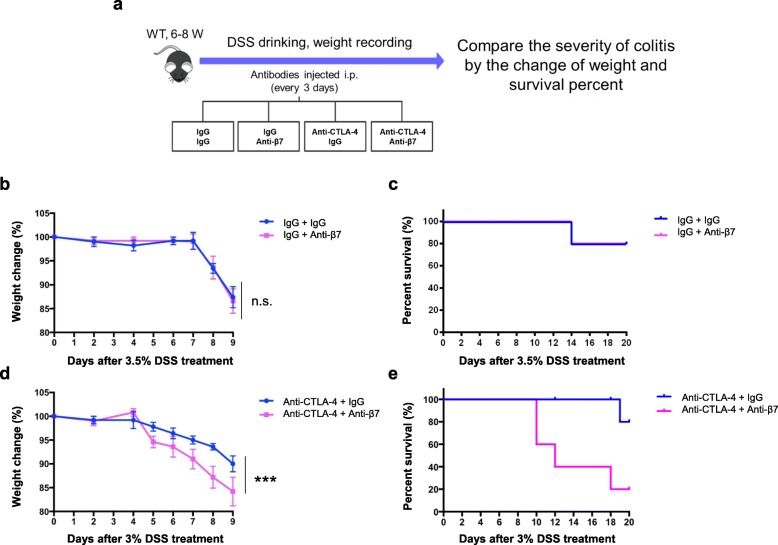
Gut-trafficking blockade increases colitis severity under the condition of checkpoint blockade. **a** The experimental design for the whole process. **b**, **c** WT mice were given 3.5% DSS for 7 days and treated without CTLA-4 blockade (IgG as the isotype control). Percent of the initial weight of mice receiving the IgG isotype control (Iso Ctrl) or anti-β7 mAb (**b**). Survival of the mice receiving the IgG isotype control (Iso Ctrl) or anti-β7 mAb (**c**). **d**, **e** WT mice were given 3% DSS for 7 days and treated with CTLA-4 blockade. Percent of the initial weight of mice receiving the IgG isotype control (Iso Ctrl) or anti-β7 mAb (**d**). Survival of the mice receiving the IgG isotype control (Iso Ctrl) or anti-β7 mAb (**e**). There were five mice in each group. The data are shown as the mean and SEM determined by two-way ANOVA with Sidak’s correction for multiple comparisons, ***P < 0.001. Survival was monitored for 20 days

Next, we wondered whether a T cell trafficking-blocking antibody could be used to treat colitis induced by DSS with or without CTLA-4 blockade. Here, we used the antibody clone FIB 504, which binds with β7 integrin to block gut trafficking by leukocytes in mice [30]. We separated WT mice into 4 groups, injected antibodies every 3 days as indicated in the boxes and treated the mice with DSS at the beginning (Fig. 4a). When we treated the mice with the anti-β7 antibody, gut homing by CD4^+^ T cells was blocked efficiently regardless of whether CTLA-4 blockade was employed (Additional file 4: Figure S4). We found that the differences in weight and survival between the gut-trafficking blockade group and the control group were nonsignificant without CTLA-4 blockade, which suggested that gut-trafficking blockade does not affect DSS-induced colitis directly (Fig. 4b, c, Additional file 5: Figure S5), consistent with a recent study showing that β7 deficiency does not exacerbate intestinal inflammation unless in the setting of impaired Treg function [31]. However, when we treated the mice with the anti-CTLA-4 antibody, the trafficking blockade group developed more severe colitis than the control group, showing more dramatic weight loss and a lower survival rate (Fig. 4d, e).

### Gut-trafficking blockade has different effects on different Th subsets

**Fig. 5:**
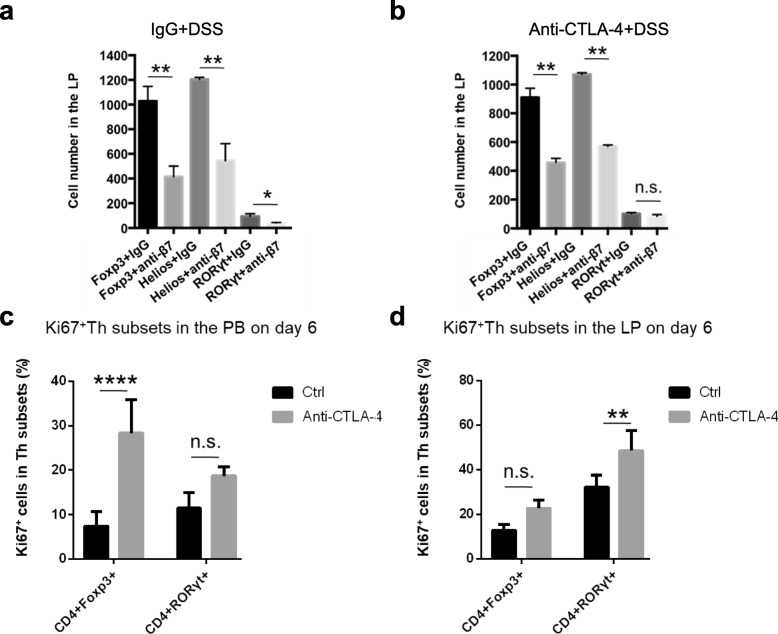
Anti-CTLA-4 antibody treatment facilitates the proliferation of Th17 cells in the LP. **a**, **b** The numbers of Tregs and Th17 cells in the LP on day 7 after treatment with the IgG antibody or anti-β7 mAb, 2.5% DSS and without (**a**) or with (**b**) CTLA-4 blockade were determined. **c**, **d** The percentages of Ki67^+^ Tregs and Th17 cells in the peripheral blood (**c**) and lamina propria (**d**) were determined 6 days after CTLA-4 blockade. There were 4-5 mice in each group. The data represent mean ± SEM. For all graphs: *P < 0.05, **P < 0.01 and ****P < 0.0001

CD4^+^ T cells are classified into functionally distinct subsets, including the Th1, Th2, Th17 and Treg subsets. To determine whether a gut-trafficking blockade antibody has different effects on different CD4^+^ T cell subsets, we analysed the number of Treg, which is an immunosuppressive subset, and inflammatory Th17 cells in the LP after DSS treatment. Without CTLA-4 blockade, when we blocked T cell homing with the anti-β7 antibody, the cell numbers in the LP were significantly decreased for both Treg and Th17 populations. This indicated that the trafficking-blocking antibody was generally effective in the context of DSS colitis (Fig. 5a). Overall colitis severity did not change due to the equal blockade of both inflammatory Th17 and immunomodulatory Treg populations (Fig. 4b). However, under the condition of CTLA-4 blockade, we found that compared to the isotype control, the anti-β7 blocking antibody profoundly decreased the number of Tregs in the LP without affecting the number of Th17 cells (Fig. 5b). Our data suggest that the trafficking-blocking antibody has a biased effect on the numbers of Tregs and Th17 cells in the inflamed intestinal tissue under different CTLA-4-blockade conditions.

### CTLA-4 blockade facilitates the proliferation of Th17 cells in the LP

Next, we investigated the reason why the treatment of trafficking blockade did not change the number of Th17 cells under CTLA-4-blockade conditions. We treated mice with the anti-CTLA-4 antibody and detected the proliferation of T cell subsets in the peripheral blood and LP. Here, we analysed the percentages of Ki67^+^ Th subsets and found that the percentage of Ki67^+^ Tregs was substantially increased in the peripheral blood after CTLA-4 blockade, while the population of Ki67^+^ Th17 cells showed no significant change (Fig. 5c), suggesting that the blood could be the main source of LP Tregs through the β7-mediated gut-homing pathway. Moreover, in the LP, CTLA-4 blockade increased the proliferation of Th17 cells without affecting the Ki67^+^ Treg population (Fig. 5d). These data indicated that the local proliferation of Th17 cells instead of trafficking from the blood might be the major contributor under CTLA-4-blockade conditions, which explained why the number of Th17 cells did not change in the LP after anti-β7 antibody treatment (Fig. 5b).

## Discussion

In our present study, we identified that CD4^+^ T cells were the primary responders in CTLA-4 blockade and that the expansion of gut-homing CD4^+^ T cells induced by anti-CTLA-4 antibodies was independent of CD103. Then, we further investigated the effect of a T cell trafficking-blocking antibody on CTLA-4 blockade-related irAEs. We used DSS-induced colitis mice as our model and chose an anti-β7 antibody to block the gut trafficking of leukocytes in the mice. Blocking T cell homing increased colitis severity under CTLA-4-blockade conditions, which contrasted with our expectation. Through further research, we found that gut-trafficking blockade had different effects on different Th subsets and could facilitate the proliferation of Th17 cells in the LP.

**Fig. 6:**
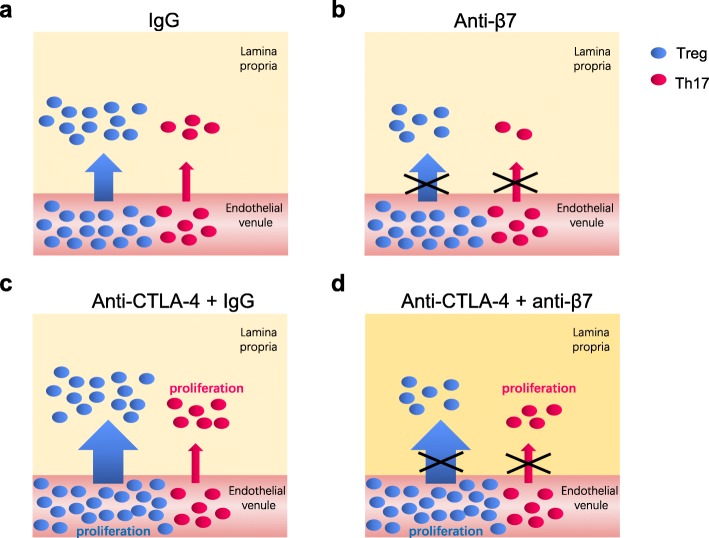
The working model for the dynamic changes in different T cell subsets under CTLA-4 and β7 blockade circumstances

In summary, activated T cells traffic to the site of inflammation in the LP in DSS-induced colitis (Fig. 6a); if we blocked the T cell homing process by injecting an anti-β7 antibody, the Treg and Th17 cell numbers in the LP both declined (Fig. 6b). These data were consistent with a previous study showing that α4β7 antibody, vedolizumab, suppressed the homing of both Teff and Treg cells from IBD patients in a humanised mouse model [32]. However, when we treated mice with an anti-CTLA-4 antibody, it promoted the proliferation of Tregs in the blood and the proliferation of Th17 cells in the LP (Fig. 6c). The majority of Tregs in the LP mainly accumulate through trafficking from the blood so if gut trafficking was blocked by the anti-β7 antibody, the number of Tregs would decrease. Since compared to that of Tregs, the number of Th17 cells in the LP was small, it was easily compensated by enhanced local proliferation, which resulted in biased trafficking-blocking effects on the numbers of Tregs and Th17 cells under the CTLA-4-blockade condition (Fig. 6d).

There are two main reasons why we chose the DSS-induced colitis model instead of the cell transfer model in this study. The first reason is that DSS oral administration is a widely used animal model to induce colitis by damaging the gut epithelia. This model is better to mimic the clinical situation that patients suffer from gut inflammation by various intestinal damages. The second reason is that although DSS-induced intestinal damage can happen in CD4 T cell depletion/deficient condition [33], the severity of colitis was dramatically decreased in Rag-1 KO mice compared with that in WT mice [34]. These results, consistent with what we previously published work [27], demonstrated T lymphocyte-related intestinal inflammations contribute significantly to the severity of DSS-induced colitis.

Our work highlights a dynamic process of homing and proliferation for functional T cell subsets in the context of immune checkpoint blockade. At present, trafficking-blocking antibodies are novel and potential drugs for IBD treatment with enhanced clinical effects and relatively few systemic adverse events [21]. Therefore, it is of great significance to illustrate the general clinical mechanism. A recent clinical report showed that seven patients who developed enterocolitis during ICPI therapy (with ipilimumab or nivolumab), six of whom had been treated with ipilimumab as a cancer therapy, were successfully treated with vedolizumab. During the treatment process, ICPI therapy was discontinued in all patients upon the development of grade 3 enterocolitis, and when response to treatment with corticosteroids indicated that these patients were either steroid refractory or steroid dependent, treatment with vedolizumab was started [25]. These clinical data combined with our results in a mouse model suggested that the timing between stopping ICPI therapy and beginning therapy with vedolizumab might be critical for efficacy. The other difference between our study and this clinical trial was that all the patients received corticosteroids before vedolizumab treatment, indicating that pre-treatment with an immunosuppressive drug might be required for the trafficking-blocking antibody to effectively reduce intestinal inflammation.

Besides, it is also worth noting that CTLA-4 may have different regulatory functions and requirements in mice and humans. Heterozygous *Ctla4* knock-out mice were reported to be phenotypically normal, whereas heterozygous *CTLA4* germline mutation will cause immune dysregulation disease in human [35, 36], which suggests that the regulatory network of CTLA-4 signalling pathway is more sensitive in human than mice.

## Conclusion

In conclusion, our data reveals the fundamental mechanism of T cells underlying trafficking-blocking antibody therapy. Our funding provides a caution for applying a trafficking-blocking antibody to treat CTLA-4  blockade-induced colitis. This work has significant implications for the clinical management of immune checkpoint therapy-induced adverse events.

## Methods

### Mice

C57BL/6 mice were purchased from Shanghai Ling Chang Biotech limited company, and CD103 KO mice were purchased from the Jackson Laboratory. For all experiments, 6- to 8-week-old female mice were used. Mice were maintained under SPF conditions in the Animal Science Centre at the Shanghai Jiao Tong University School of Medicine.

### Induction of DSS colitis and injection of antibody

Mice received 2–4% DSS (MP Biomedicals) in their drinking water for 6–7 days. Weight was recorded daily. Prepare the antibody (anti-CTLA-4 mAb, clone 9D9, BioXCell; anti-β7, clone FIB504, BioXCell and isotype control, BioXCell) solution with PBS to 1 μg/μl, intraperitoneal injection with 200 μg per mouse every 3 days.

### Histological analysis

Colon tissues were fixed with 4% paraformaldehyde and stained with haematoxylin and eosin. Each sample was given a score of 0–4 based on the following criteria: (1) severity of inflammation, (2) percent of area affected by inflammation, (3) degree of hyperplasia, (4) percent of area affected by hyperplastic changes and (5) ulceration.

### Serum cytokine measurement

Blood samples were collected at the last day (day 47) of the whole process. After clotting at least 30 min at room temperature, the serum was separated with centrifuge (10 min at 1200 relative centrifugal force). Luminex assay was performed following the product manual.

### LP isolation

Sacrifice the mice, cut off the colons and remove the remaining fat tissue and the Peyer’s patches first. Then cut longitudinally and wash in PBS. Transfer the colon into solution A (1 mM DTT, 30 mM EDTA, 10 mM HEPES) in order to remove epithelial cells. Transfer the colon into solution B (30 mM EDTA, 10 mM HEPES) to remove the remaining DTT. Cut the colon into small sections and incubate in digest solution (collagenase VIII and DNase I: freshly add to 1640 Complete Medium, 1: 600) and put it at 37 °C in a 5% CO_2_ incubator for 55 min. Following the digestion step, pass the tissue through 100-μm cell strainers, centrifuge and resuspend the cell pellet with 4 ml 40% Percoll, mix thoroughly and carefully underlay 2.5 ml 80% Percoll at the bottom of the 40% Percoll solution. Centrifuge 2500 rpm, 25 min at RT (Acceleration 1, deceleration 1). Carefully harvest the mononuclear cells from the interphase of the 80% and 40% Percoll gradient with Pasteur pipette, add cold PBS, mix thoroughly with Pasteur pipette and centrifuge 800 *g*, 10 min at 4 °C. Resuspend the pellets with PBS and pass through a 70-μm filter. Centrifuge again, then resuspend the cells with FACS buffer (0.5% BSA-PBS) and counting.

### Peripheral blood cell isolation

Collect mice blood from the retro-orbital plexus, and use 2 mM EDTA to prevent clotting. Filter through a 40-μm filter, wash once with PBS and then prepare for the following staining step.

### Flow cytometry

Cell surface antigen staining has used the following antibody conjugates in this study: anti-CD4 (clone RM4-5, Biolegend), anti-CD8 (clone 53-6.7, Biolegend), anti-CD44 (clone IM7, BD Biosciences), anti-62 L (clone MEL-14, Biolegend), anti-β7 (clone FIB504, Biolegend), anti-α4 (clone R1-2, Biolegend), Rat IgG2b,κ Iso control (clone eB149/10H5, eBioscience), Rat IgG2a, κ Iso control (clone eBR2a, eBioscience) and LIVE/DEAD Aqua (Thermofisher), and was performed in the dark with antibody dilutions in FACS buffer (0.5% BSA-PBS) for 30 min at 4 °C. Cells were washed twice with FACS buffer, resuspended in FACS buffer and acquired or used for subsequent intracellular staining.

As for intracellular staining, after cell surface staining, cells were permeabilized by the fixation/permeabilization reagents (eBioscience) in the dark for 45 min at room temperature, then washed twice with permeabilization buffer and used for intracellular staining; the permeabilization process followed the specification.

Intracellular staining was performed in the dark at 4 °C with antibody dilutions in FACS buffer for 60 min and has used the following antibodies: anti-RORγt (clone AFKJS-9, invitrogen), anti-Foxp3 (clone FJK-16 s, eBioscience), anti-Helios (clone 22F6, eBioscience) and anti-Ki67 (clone B56, BD Biosciences). All flow cytometry analyses were performed on an LSRFortessa flow cytometer (BD Biosciences) and data were analysed with FlowJo X.

### Statistical analysis

All values are shown as mean ± SEM. Statistical significance was determined by Student’s *t* test. Statistical analysis was done with GraphPad Prism 7.

## Supplementary information

**Additional file 1: Figure S1.** α4^+^CD44^+^ blood CD4^+^ T cell numbers increase after CTLA-4 blockade. Flow cytometric analysis of gut-homing CD4^+^ T cells in the blood 10 days after isotype antibody (Ctrl) or anti-CTLA-4 antibody treatment.

**Additional file 2: Figure S2.** Isotype controls of anti-β7 and anti-CD44 antibodies. Flow cytometric analysis of blood gut-homing CD4^+^ and CD8^+^ T cells collected from WT mice. Isotype control staining is shown as in the left panels. Isotype control for β7 antibody is Rat IgG2a, κ and isotype control for CD44 antibody is Rat IgG2b, κ.

**Additional file 3: Figure S3.** CD4^+^ subset exhibits a higher CTLA-4 expression level than the CD8^+^ subset. Gene expression data from the Immunological Genome for *Ctla4* in reference population.

**Additional file 4: Figure S4.** Anti-β7 antibody blocks gut-homing CD4^+^ T cells in the blood. Flow cytometric analysis of gut-homing CD4^+^ T cells (β7^+^CD44^+^) 10 days after IgG (isotype control) or anti-CTLA-4 antibody treatment with or without β7 blockade (IgG as the isotype control).

**Additional file 5: Figure S5.** Gut-trafficking blockade does not affect 3% DSS-induced colitis directly. WT mice treated with the IgG isotype control (Iso Ctrl) or anti-β7 mAb without CTLA-4 blockade (IgG as the isotype control), and given 3% DSS for 7 days. **a** Percent of the initial weight of mice receiving the IgG isotype control (Iso Ctrl) or anti-β7 mAb. **b** Survival of the mice receiving the IgG isotype control (Iso Ctrl) or anti-β7 mAb. 5 mice in each group. The data are shown as the mean and SEM determined by two-way ANOVA with Sidak’s correction for multiple comparisons. Survival was monitored for 20 days.

## Data Availability

All data generated or analysed during this study are included in this published article and its supplementary information files.

## Abbreviations

CTLA-4: Cytotoxic T lymphocyte-associated antigen-4
DSS: Dextran sulfate sodium
FDA: Food and Drug Administration
GALT: Gut-associated lymphoid tissue
irAEs: Immune-related adverse events
ICPI: Immune checkpoint inhibitor
IBD: Inflammatory bowel disease
LP: Lamina propria
MAdCAM-1: Mucosal addressin cell adhesion molecule-1
MLN: Mesenteric lymph node
PPs: Peyer’s patches
WT: Wild-type
