# Supplementary figures and images for: The role of T cell trafficking in CTLA-4 blockade-induced gut immunopathology
Source: BMC Biol. 2020 Mar 17;18:29. doi: 10.1186/s12915-020-00765-9 (PMC7079427; doi:10.1186/s12915-020-00765-9)

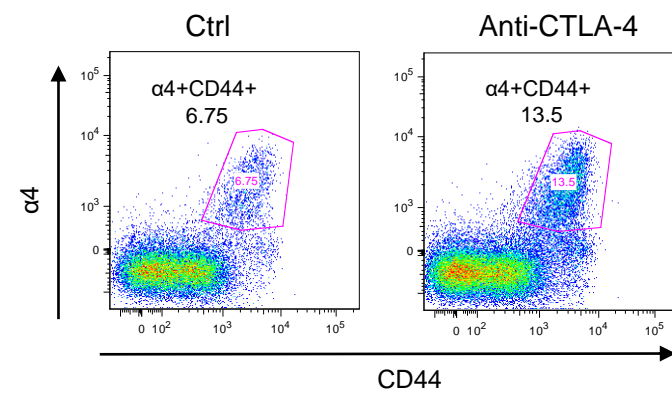

Supplement: Supplementary file 1 — Additional file 1: Figure S1. α4+CD44+ blood CD4+ T cell numbers increase after CTLA-4 blockade. Flow cytometric analysis of gut-homing CD4+ T cells in the blood 10 days after isotype antibody (Ctrl) or anti-CTLA-4 antibody treatment. [file 12915_2020_765_MOESM1_ESM.pdf]

**a**

Gate on CD4+ subset

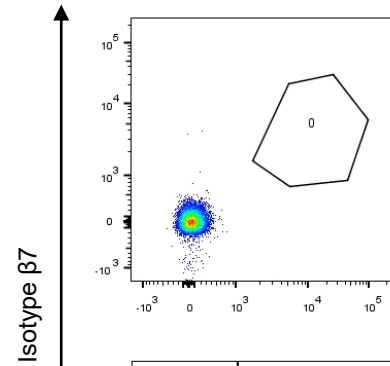

**b**

Gate on CD8+ subset

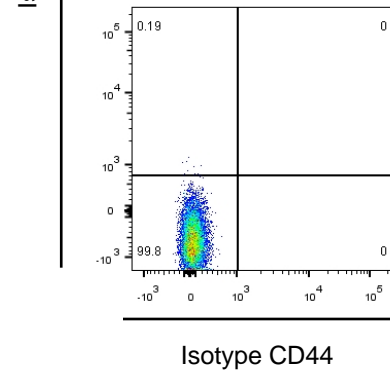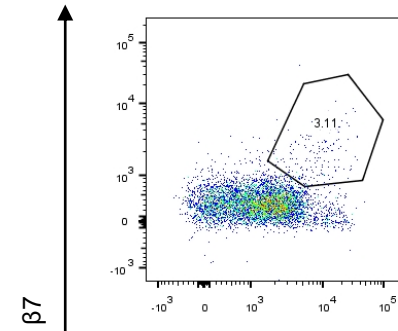

$\beta 7$

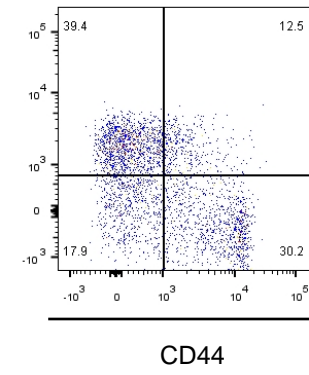

CD44

Supplement: Supplementary file 2 — Additional file 2: Figure S2. Isotype controls of anti-β7 and anti-CD44 antibodies. Flow cytometric analysis of blood gut-homing CD4+ and CD8+ T cells collected from WT mice. Isotype control staining is shown as in the left panels. Isotype control for β7 antibody is Rat IgG2a, κ and isotype control for CD44 antibody is Rat IgG2b, κ. [file 12915_2020_765_MOESM2_ESM.pdf]

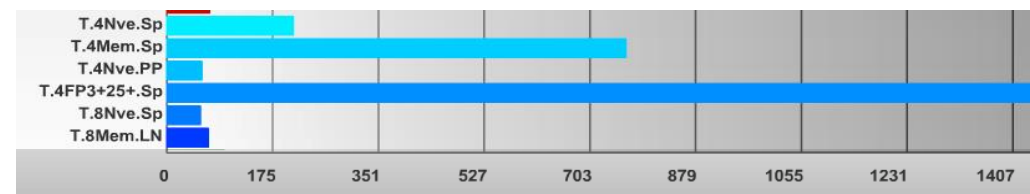

Supplement: Supplementary file 3 — Additional file 3: Figure S3. CD4+ subset exhibits a higher CTLA-4 expression level than the CD8+ subset. Gene expression data from the Immunological Genome for Ctla4 in reference population. [file 12915_2020_765_MOESM3_ESM.pdf]

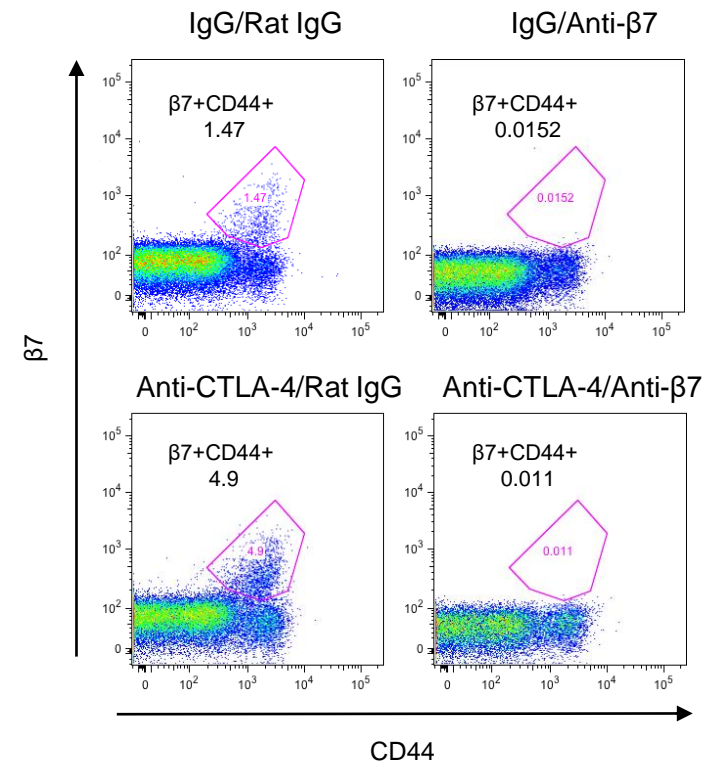

Supplement: Supplementary file 4 — Additional file 4: Figure S4. Anti-β7 antibody blocks gut-homing CD4+ T cells in the blood. Flow cytometric analysis of gut-homing CD4+ T cells (β7+CD44+) 10 days after IgG (isotype control) or anti-CTLA-4 antibody treatment with or without β7 blockade (IgG as the isotype control). [file 12915_2020_765_MOESM4_ESM.pdf]

**a**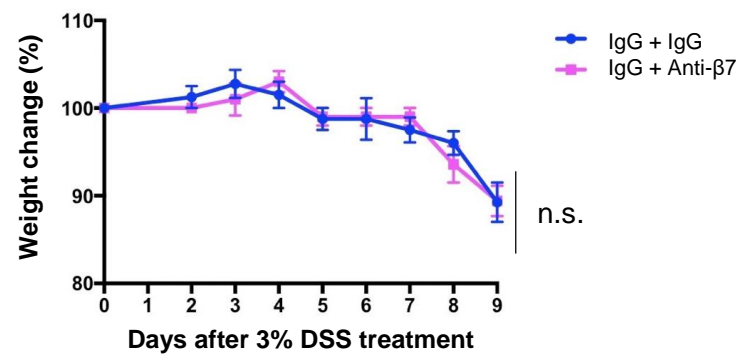**b**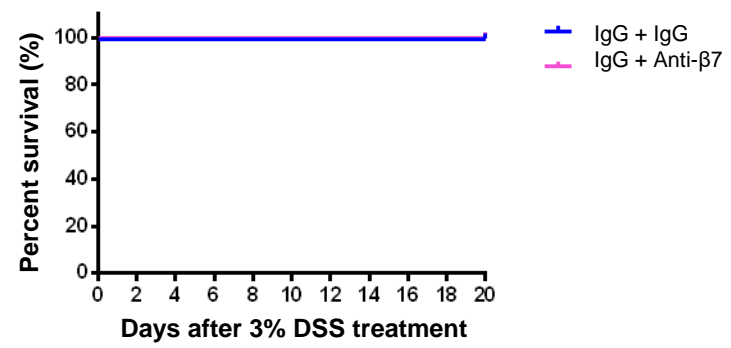

Supplement: Supplementary file 5 — Additional file 5: Figure S5. Gut-trafficking blockade does not affect 3% DSS-induced colitis directly. WT mice treated with the IgG isotype control (Iso Ctrl) or anti-β7 mAb without CTLA-4 blockade (IgG as the isotype control), and given 3% DSS for 7 days. a Percent of the initial weight of mice receiving the IgG isotype control (Iso Ctrl) or anti-β7 mAb. b Survival of the mice receiving the IgG isotype control (Iso Ctrl) or anti-β7 mAb. 5 mice in each group. The data are shown as the mean and SEM determined by two-way ANOVA with Sidak’s correction for multiple comparisons. Survival was monitored for 20 days. [file 12915_2020_765_MOESM5_ESM.pdf]
